# Effect of N-Acetylcysteine on Residual Renal Function in Chronic Haemodialysis Patients Treated with High-Flux Synthetic Dialysis Membranes: A Pilot Study

**DOI:** 10.5402/2013/636208

**Published:** 2012-11-26

**Authors:** Leonid Feldman, Ramzia Abu Hamad, Shai Efrati, Ali Ashker, Ilia Beberashvili, Michal Shani

**Affiliations:** ^1^Nephrology Division and Research & Development Unit, Assaf Harofeh Medical Center, Zerifin 70300, Israel; ^2^Sackler Faculty of Medicine, Tel Aviv University, Tel Aviv 69978, Israel; ^3^Family Medicine Department, Clalit Health Services and Faculty of Medicine, Hebrew University of Jerusalem, Jerusalem 97904, Israel

## Abstract

*Background*. Preservation of residual renal function in chronic dialysis patients has proven to be a major predictor of survival. The aim of the present study was to investigate an ability of the combined use of N-acetylcysteine and high-flux biocompatible haemodialysis membranes to improve residual renal function in haemodialysis patients. *Patients and Methods*. Chronic haemodialysis patients with a residual urine output of at least 100 mL/24 h were administered oral an N-acetylcysteine 1200 mg twice daily for 2 weeks. Treatment group included patients treated with dialysers using high-flux synthetic biocompatible membranes. Control group included patients treated with dialysers using low-flux semisyntetic triacetate haemodialysis membranes. *Results*. Eighteen patients participated in the study. The residual glomerular filtration rate showed a nonsignificant trend for increase in both groups. The magnitude of GFR improvement after N-acetylcysteine administration was less pronounced in the group treated with high-flux biocompatible membranes: +0.17 ± 0.56 mL/min/1.73 m^2^ in treatment group and +0.65 ± 0.53 mL/min/1.73 m^2^ in control group (*P* < 0.05). *Conclusion*. In this study of favorable effect of N-acetylcysteine on residual renal function in chronic haemodialysis patients may be less pronounced when using high-flux biocompatible, rather than low-flux semisyntetic, HD membranes.

## 1. Introduction 

Preserving residual renal function (RRF) has always been the primary clinical goal for every nephrologist managing patients with chronic kidney disease. RRF in patients of dialysis has proven to be a consistent and powerful predictor of mortality [1, 2]. This is particularly evident for patients treated with peritoneal dialysis (PD): CANUSA study showed that only RRF but not dialysis dose has significant predictive power of mortality [1].

Up till now, there have been a very few studies that have examined the contribution of RRF to outcome in haemodialysis (HD) patients [2–4]. This is particularly unfortunate because HD patients count at least 93% of all dialysis population in many different countries, such as Portugal, USA, and Israel [5]. Moreover, it is well known that RRF deteriorates more rapidly in HD patients than in PD patients [6]. Preservation of RRF remains an unresolved problem in dialysis patients. 

Nevertheless, several important facts may provide some hope for better maintenance of RRF in HD patients. First, using modern membranes for haemodialysis (specifically—high flux biocompatible membranes) may lead to a significantly better preservation of RRF [7]. Second, in our recent studies, we show that N-acetylcysteine (NAC) may improve RRF in prevalent HD and PD patients [8, 9]. 

The aim of our study is to investigate an ability of antioxidant therapy by NAC combined with the usage of high-flux HD membranes to produce an additional favourable effect on RRF in prevalent HD patients.

## 2. Subjects and Methods

The study included 18 adult patients with end-stage renal disease treated with chronic HD at Assaf Harofeh Medical Center. The study was approved by the Ethical Committee of Assaf Harofeh Medical Center and all patients signed an informed consent before their inclusion in the study. Patients were excluded from the study if they were treated with chronic dialysis for less than 3 months, had acute renal failure, were currently treated with antioxidants, such as N-acetylcysteine (NAC), vitamins E, C, and herbal medications, or were anuric (less than 100 mL urine in 24 h). 

The patients were divided into 2 groups. For the treatment group 6 patients were prospectively recruited from the patients treated with “ELISIO-19H” dialysers using high-flux biocompatible membranes (material—*POLYNEPHRON*, manufactured by NIPRO Co., Kita-Ku, Osaka, Japan). Data on 12 patients for control group was obtained from the database of our recent study on NAC influence on RRF [8]. These patients were treated with “SUREFLUX-190” dialyzers using low-flux semisyntetic triacetate dialysis membranes (NIPRO Co., Kita-Ku, Osaka, Japan). Both types of dialysers have the same membrane surface area of 1.9 m² and similar characteristics of small molecules clearance. 

All the patients intentionally continued their previous treatment regimen, dialysis prescription, and blood pressure medication use, including ACE-inhibitors and diuretics. All patients received an oral dose of NAC 1200 mg twice daily for 2 weeks and underwent investigation of RRF at baseline and at the end of this therapy. Blood pressure was recorded and blood samples were collected before initial and final dialysis sessions. Adequacy of hemodialysis was estimated using fractional clearance of urea as a function of its distribution volume (*Kt/V*) and was determined by the *Kt/V* natural logarithm formula [10]. 

RRF was assessed through a midweek interdialytic urine collection for the measurement of urine output and calculation of residual renal *Kt/V* and glomerular filtration rate (GFR). Patients emptied their bladders before dialysis. Urine output was expressed as mL per 24 h. Blood for urea and creatinine was sampled at the end of the current and at the start of the next dialysis session. Residual GFR was measured as the mean of urea and creatinine clearance normalized to 1.73 m^2^ body surface area [11]. The residual renal *Kt/V* was calculated as the renal urea clearance per week adjusted for the urea distribution volume [4].

The serum level of nitric oxide (NO) was measured using a commercially available enzymatic assay (R&D Systems Europe Ltd., Abingdon, UK). Serum levels of asymmetric dimethylarginine (ADMA) were measured by a commercially available enzyme immunoassay (ADMA-ELISA, DLD Diagnostika, Hamburg, Germany).

Compliance with oral therapy using NAC was ascertained by pill counts once weekly during dialysis sessions. 

### 2.1. Statistical Analysis

The data for studied variables was collected before and after therapeutic intervention with NAC. Comparisons of pretreatment and posttreatment mean values were made with nonparametric Wilcoxon rank-sum test. This analysis was conducted using STATA 8.0 statistical software (StataCorp, College Station, TX, USA). *P* values < 0.05 were considered significant. Results are expressed as mean ± standard deviation (SD). 

## 3. Results

The demographic and clinical data of the patients are presented in Table 1. Eighteen patients participated in the study. Mean duration of dialysis therapy was 2–2.5 years. Baseline dialysis urea clearance was adequate with mean dialysis *Kt/V* 1.33-1.34 per session. 

Changes in patients' clinical and laboratory characteristics between baseline and 2-week treatment are shown in Table 2. The RRF showed some nonsignificant trend for improvement during the study period in both groups of patients. The residual GFR increased from 1.61 ± 1.36 to 1.78 ± 1.59 mL/min/1.73 m² in treatment group and from 1.53 ± 0.88 to 2.18 ± 1.12 mL/min/1.73 m² in control group. The magnitude of GFR improvement after NAC administration was significantly less pronounced in the group treated with HF biocompatible membranes than in group treated with semisynthesis membranes: +0.17 ± 0.56 mL/min/1.73 m² in treatment group and +0.65 ± 0.53 mL/min/1.73 m² in control group (*P* < 0.05). Mean 24-hours urine volume and residual renal *Kt/V* per week calculation showed a non-significant increase in both groups (Table 2). There were no statistically significant changes in serum levels of NO and ADMA.

NAC was well tolerated and no adverse events, such as allergy or intradialytic hypotensive episodes, developed during the treatment. 

## 4. Discussion 

In this study the use of NAC together with biocompatible membranes for haemodialysis was not shown to provide an additional favourable effect on RRF. Although the positive effect of NAC on RRF was demonstrated both in patients dialysed with synthetic biocompatible and semisyntetic membranes, this action was more pronounced in the later group. 

The demonstrated augmentation in residual GFR in the control group, as well as the difference between two study groups (0.65 and 0.48 mL/min/1.73 m², consequently) seems to be small, but in fact it may be associated with significant clinical benefits. A reanalysis of the CANUSA study showed a 12% decrease in the relative risk of death for each 5 liter per week per 1.73 m^2^ increase in residual GFR (which corresponds to about 0.5 mL/min/1.73 m^2^) [1]. 

The absence of possible additive beneficial effect of biocompatible membranes and NAC on RRF in our study is not easily explained. Although hypotensive episodes during haemodialysis may lead to more rapid loss of RRF [6], they were avoided in our study.

Several experimental and clinical studies showed that increased oxidative stress in dialysis patients may lead to the deterioration of RRF [6]. Based on current knowledge, treatment aimed at reducing oxidative stress should be beneficial [12], and it is logical to suggest that such a therapy might help to preserve RRF in HD patients. Using high-flux HD membranes had been shown to be effective in reducing inflammatory reaction in HD patients [7]. 

Although our results do not prove the hypothesis that simultaneous use of biocompatible membranes and NAC may exert additive positive effect on RRF; at least two facts are encouraging: first, beneficial effect of NAC on RRF and second, association of both types of membranes used with favorable effect on RRF.

N-Acetylcysteine (NAC) is an active antioxidant proved to be safe and beneficial in haemodialysis patents [12]. In our recently completed study, NAC effectively improved residual renal function in chronic haemodialysis patients [8]. 

 In several experimental models, NAC has been shown to exert a vasodilatory effect on renal microcirculation [13]. It is thought that the favorable effect of NAC is mediated, at least in part, by its antioxidant properties. In addition, some studies suggest that NAC may ameliorate renal ischemia-reperfusion injury, apparently promoting a vasodilatory effect [13]. Vascular reactivity in dialysis patients is associated with plasma concentrations of NO and ADMA [14]. NAC had been previously shown to be able to decrease plasma level of ADMA in HD patients [15]. In our present study, we were unable to demonstrate any ability of NAC to influence serum levels of NO and ADMA. 

Our study has several limitations, necessitating a cautious approach to the conclusions. Firstly, this is a single-centre pilot trial with a small cohort of patients. Secondly, this study was short term. Thirdly, we were able to involve only prevalent HD patients with considerable dialysis vintage. Further studies are needed in a larger cohort of incident dialysis patients and a prolonged period of treatment. 

In conclusion, our data suggest that the favorable effect of short term treatment with NAC on RRF in chronic HD patients may be less pronounced when using HF biocompatible, rather than semisyntetic HD membranes. 

## Figures and Tables

**Table 1:** Demographic and clinical characteristics of participants, mean (±SD).

	Treatment group	Control group
Number of patients	6	12
Age, years	63.5 ± 12.6	70.5 ± 10.3
Gender, male	5 (83%)	10 (85%)
Vintage on dialysis, months	24.3 ± 15.9	30.2 ± 26.4
Arteriovenous fistula as dialysis access, patients	5 (83%)	9 (75%)
Systolic blood pressure, mmHg	140.8 ± 18.7	136.0 ± 19.1
Diastolic blood pressure, mmHg	71.3 ± 16.8	70.2 ± 16.2
Dialysis adequacy (*Kt*/*V*, session)	1.33 ± 0.22	1.34 ± 0.15
Haemoglobin, g/dL	11.6 ± 1.6	11.6 ± 1.1
Albumin, g/L	38.7 ± 3.9	40.2 ± 2.9
CRP	13.7 ± 17.1	13.5 ± 15.6
Cause of renal disease, patients		
Diabetes mellitus	5 (83%)	7 (58%)
Hypertension	1 (17%)	2 (17%)
ACE inhibitors or ARBs use, patients	3 (50%)	6 (50%)
Furosemide use, patients	4 (67%)	7 (58%)

**Table 2:** Effect of N-acetylcysteine on clinical characteristics and residual renal function, mean (±SD).

	Treatment group		Control group	
	Baseline	NAC therapy	Baseline	NAC therapy
Predialysis weight, kg	75.2 ± 13.1	75.7 ± 12.9	82.1 ± 14.9	82.2 ± 15.3
Systolic BP, mmHg	140.8 ± 18.1	142.7 ± 12.2	136.0 ± 19.1	138.2 ± 18.3
Diastolic BP, mmHg	71.3 ± 16.8	73.7 ± 16.2	70.7 ± 16.2	69.3 ± 12.9
Ultrafiltration on dialysis session, L	2.7 ± 0.26	2.4 ± 0.62	2.6 ± 0.89	2.7 ± 0.86
Urine volume, mL/24 h	268 ± 174	313 ± 185	366 ± 203	497 ± 242
*Kt*/*V* renal (per week)	0.25 ± 0.23	0.32 ± 0.24	0.21 ± 0.11	0.33 ± 0.14
Residual GFR, mL/min/1.73 m²	1.61 ± 1.36	1.78 ± 1.59	1.53 ± 0.88	2.18 ± 1.12
Residual GFR change, mL/min/1.73 m²	NA	+0.17 ± 0.56	NA	+0.65 ± 0.53*
Serum NO, mcmol/L	35.1 ± 28.6	34.6 ± 15.0	61.8 ± 68.5	58.2 ± 60.4
Serum ADMA, mcmol/L	0.54 ± 0.24	0.53 ± 0.19	0.91 ± 0.20	0.93 ± 0.18

NAC: N-acetylcysteine; BP: blood pressure; GFR: glomerular filtration rate; NO: nitric oxide; ADMA: asymmetric dimethylarginine.

*P < 0.05 versus corresponding value.

NA: not applicable.
